# Intraoperative Patient Warming Instead of Gas on the Management of Postoperative Pain in Laparoscopic Colectomy and Cholecystectomy: A Randomized Controlled Trial

**DOI:** 10.7759/cureus.57989

**Published:** 2024-04-10

**Authors:** Fatma Alkan Bayburt, Guniz Meyanci Koksal, Azime Bulut, Ilker Sengul

**Affiliations:** 1 Anesthesiology and Reanimation, Giresun Education and Research Hospital, Giresun, TUR; 2 Anesthesiology and Reanimation, Istanbul University Faculty of Medicine, Istanbul, TUR; 3 Anesthesiology and Reanimation, Giresun University Faculty of Medicine, Giresun, TUR; 4 Endocrine and General Surgery, Giresun University Faculty of Medicine, Giresun, TUR

**Keywords:** general surgery, surgery, anesthetic agent, anesthesia, laparoscopic cholecystectomy, laparoscopic colectomy, postoperative pain, pain, warming, intraoperative

## Abstract

Introduction: Blurred lines remain in details of the association between patient warming with postoperative pain and a proper analgesic requirement. Therefore, the present study proposes to observe the effects of intraoperative patient warming and carbon dioxide insufflation duration in laparoscopic colectomy (LCol) and laparoscopic cholecystectomy (LChol) procedures on postoperative pain, analgesic requirements, and hemodynamics.

Methods: The present study involved 80 cases aged 18-80 years with the American Society of Anesthesiologists I-III classification, possessing two initial groups primarily, one for LCol and one for LChol. Subsequently, each was divided into two through randomization for intraoperative warming. Postoperatively, pain perception, *per se*, was evaluated using the visual analog scale (VAS) score at the 30 minutes, 1st, 6th, 12th, and 24th hours, along with the impact of pain on hemodynamic parameters and side effects such as nausea/vomiting and the dosage of analgesics used.

Results: Groups actively heated with warm air-blowing devices detected significantly higher intraoperative core and skin temperature measurements, and postoperative early pain perception was significantly lower in the warmed ones. Furthermore, a significant decrease in the VAS scores and the analgesic at the 12th and 24th hours compared to the first six hours was recognized between them.

Conclusion: Consequently, herewith, we postulate that so-called patient warming positively affects the VAS scores.

## Introduction

Intraoperative hypothermia, *per se*, is defined as a decrease in core body temperature to below 36°C. It is known that the core temperature drops in the first 30 minutes after anesthesia induction. Thus, it is recommended to perform temperature monitoring for patients undergoing surgery lasting longer than 30 minutes [1]. Briefly, heat loss depends on the wound site, which can be more significant compared to laparoscopy in conventional surgical procedures. However, alterations in temperature due to carbon dioxide (CO2) insufflation during laparoscopic surgeries appear [2-6]. In addition, warming and humidification of insufflated CO2 gas have been reported in order to reduce the development of hypothermia [7-11].

As such, the present study aimed to observe the effects of the duration of the intraperitoneal gas insufflation in laparoscopic colectomy (LCol) and laparoscopic cholecystectomy (LChol), the intraabdominal pressure level, and the method of warming on postoperative pain, analgesic consumption, and hemodynamics.

## Materials and methods

The study was conducted on 80 patients who had undergone LCol and LChol electively. To this end, we included patients aged 18-65 years with the American Society of Anesthesiologists stage I-II in this prospective, randomized, double-blinded study. The principal researcher visited the patients one day before surgery. The study protocol was explained to them, and information was provided about the visual analog scale (VAS). Herein, written consent was obtained from all patients. The present study was approved by the Local Ethics Committee of Istanbul University (approval number: 2011.11.21/7592).

Study population and randomization

A total of 130 patients were evaluated for the study. Moreover, 10 cases refused to participate in the survey, while 10 required open surgery in the LChol group. In the colectomy group, five patients were excluded from the study due to hemodynamic instability. Patients were randomized into two groups (n = 20): the cases were to be warmed (LCol warmed (LColw) and LChol warmed (LCholw)) and monitored at room temperature (LCol room temperature (LColrt) and LChol room temperature (LCholrt)) with a random computer program for both surgery groups. Of note, the leading researcher randomized and performed the interventions until the end of surgery. Postoperative data was recorded by the researcher blinded to the patient. The skin temperature was measured and recorded with an axillary probe, and the core temperature was measured and recorded through the nose with an esophageal probe after providing standard monitoring conditions for each.

Intraoperative follow-up and extubation

Patients were premedicated with intravenous midazolam at a dose of 0.03 mg/kg. Following routine monitoring, all patients received general anesthesia with propofol, fentanyl, and rocuronium. The room temperature was monitored at 22-24°C in all the above-mentioned groups. The purpose of the study was to obtain a body temperature of 37°C in the group that we warmed, and the cases were warmed with an air-blowing device. All blood and its products and fluids over 1000 mL were administered by warming them to 37°C with the intravenous heating system. The pressure of CO2 gas insufflated into the abdomen was recorded throughout the surgery. Prior to extubation, all patients received subcutaneous local anesthesia at the trocar entry incision site. Before the extubation process, we administered metamizole sodium (3 g) to all the cases in 100 mL serum physiologic supply and reversed rocuronium with sugammadex.

Postoperative data collection

All the patients were evaluated at their postoperative 30th minute, 1st, 6th, 12th, and 24th hours after the extubation process, and their vital parameters and VAS were recorded by an anesthesiologist who blinded the patient. One gram of metamizole sodium was administered intravenously to the cases with VAS≥4, and 1 mg/kg of tramadol was distributed to the ones with VAS≥4 after metamizole injection. The primary outcome of the study was to determine the effect of warming on the postoperative VAS values.

Statistical analysis

Statistical analyses were performed using the Number Cruncher Statistical System (NCSS) 2007 and 2008 Statistical Software (Utah, USA). Descriptive statistical methods (mean, standard deviation, median, frequency, and ratio) were used for data evaluation. A one-way analysis of variance test was used for normally distributed data, and a Kruskal-Wallis test was used for parameters that did not show a normal distribution. The Mann-Whitney U test was used to determine the group causing the difference. For group comparisons of parameters that did not show normal distribution, the Friedman for the comparison of qualitative data, the Pearson chi-square test, and the Fisher Freeman Halton test were used, and for determining the group causing the difference, the Fisher's exact test and Yates' continuity correction test were used. Significance was evaluated at p<0.01 and p<0.05 levels.

## Results

**Figure 1:**
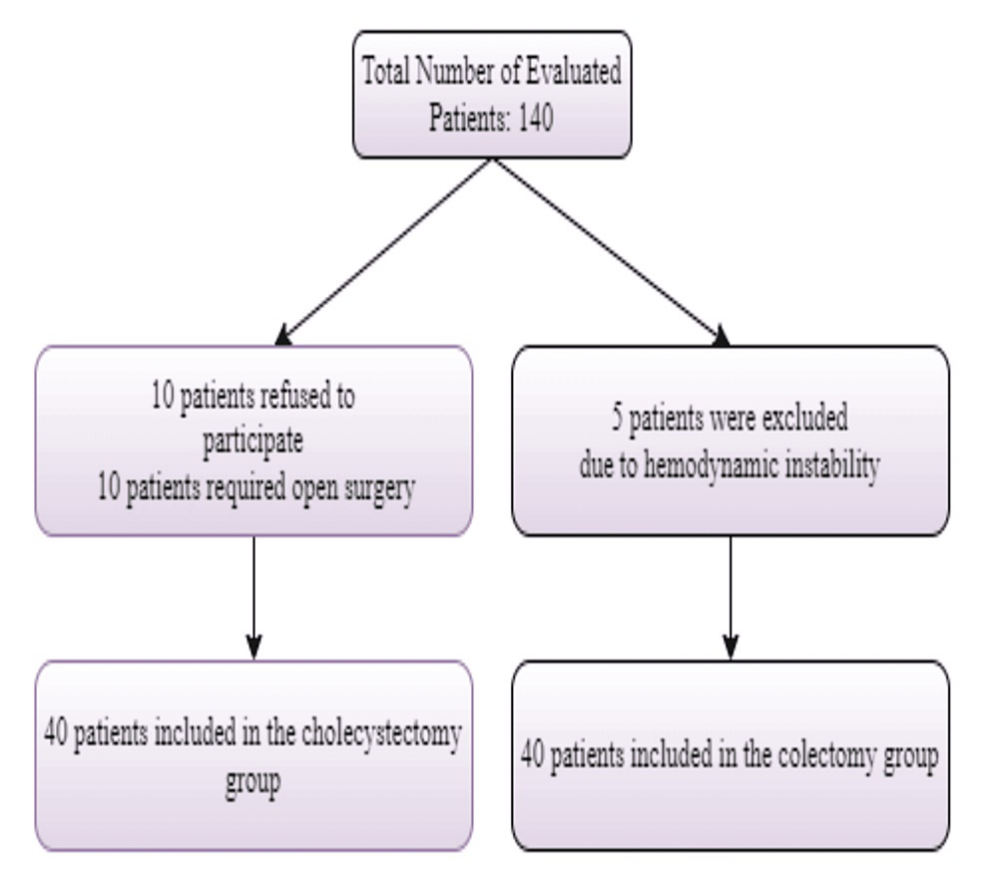
CONSORT structure of the studied population CONSORT: Consolidated Standards of Reporting Trials

We evaluated a total of 130 patients, including 80 cholecystectomy patients and 50 colectomy patients, before the study. After applying our exclusion criteria, we found 60 eligible patients in the cholecystectomy group. Ten patients refused to participate, and ten patients required open surgery, so they were excluded from the study. In total, 40 patients were included in the study. In the colectomy group, 45 patients were eligible for the study. Five patients were excluded due to hemodynamic instability, resulting in 40 patients included in the study. As such, our study was conducted on 40 LChol cases and 40 cases of LCol. No significant difference was recognized while they were evaluated in terms of age, body mass index, gender, and CO2 insufflation pressure (p>0.05). Besides, the duration of surgery and CO2 insufflation were similar, while the patients’ core and axillary temperature levels during surgery were high in warmed cases, as expected. The VAS levels in the 30th minute of LChol were higher than in LCol (p=0.018, p<0.05). However, the VAS in the 1st and 12th hours of LChol were significantly lower than LCol (p<0.05). All the warmed cases had significantly lower VAS levels at the 30th minute, 1st hour, and 6th hour than the others. In postoperative vital signs, the systolic blood pressure at the 30th minute, 1st hour, and 6th hour of LCholrt was lower than the other groups. No statistically significant differences were observed in the respiratory and heart rate measurements at all times. Finally, no differences were detected between the groups regarding postoperative nausea and vomiting (Tables 1-4, Figure 1).

**Table 1:** CONSORT 2010 checklist of information to include when reporting a randomized trial CONSORT: Consolidated Standards of Reporting Trials

Section/topic	Item No	Checklist item	Reported on page no
Title and abstract			
	1a	Identification as a randomized trial in the title	1
	1b	Structured summary of trial design, methods, results, and conclusions (for specific guidance, see CONSORT for abstracts)	1
Introduction			
Background and objectives	2a	Scientific background and explanation of the rationale	1
	2b	Specific objectives or hypotheses	1
Methods			
Trial design	3a	Description of trial design (such as parallel, factorial) including allocation ratio	1
	3b	Important changes to methods after trial commencement (such as eligibility criteria), with reasons	1-2
Participants	4a	Eligibility criteria for participants	1
	4b	Settings and locations where the data were collected	2
Interventions	5	The interventions for each group with sufficient details to allow replication, including how and when they were actually administered	2
Outcomes	6a	Completely defined pre-specified primary and secondary outcome measures, including how and when they were assessed	2
	6b	Any changes to trial outcomes after the trial commenced, with reasons	NA
Sample size	7a	How sample size was determined	2
	7b	When applicable, explanation of any interim analyses and stopping guidelines	NA
Randomization:			
Sequence generation	8a	The method used to generate the random allocation sequence	2
	8b	Type of randomization; details of any restriction (such as blocking and block size)	2
Allocation concealment mechanism	9	The mechanism used to implement the random allocation sequence (such as sequentially numbered containers), describing any steps taken to conceal the sequence until interventions were assigned	2
Implementation	10	Who generated the random allocation sequence, who enrolled participants, and who assigned participants to interventions	2
Blinding	11a	If done, who was blinded after assignment to interventions (for example, participants, care providers, those assessing outcomes), and how	2
	11b	If relevant, a description of the similarity of interventions	NA
Statistical methods	12a	Statistical methods used to compare groups for primary and secondary outcomes	2
	12b	Methods for additional analyses, such as subgroup analyses and adjusted analyses	2
Results			
Participant flow (a diagram is strongly recommended)	13a	For each group, the number of participants who were randomly assigned received the intended treatment and were analyzed for the primary outcome	2-3
	13b	For each group, losses and exclusions after randomization, together with reasons	2-3
Recruitment	14a	Dates defining the periods of recruitment and follow-up	NA
	14b	Why the trial ended or was stopped	NA
Baseline data	15	A table showing baseline demographic and clinical characteristics for each group	Table 1
Numbers analysed	16	For each group, the number of participants (denominator) included in each analysis and whether the analysis was by originally assigned groups	3-4
Outcomes and estimation	17a	For each primary and secondary outcome, results for each group, and the estimated effect size and its precision (such as 95% confidence interval)	Table 2 and Table 3
	17b	For binary outcomes, presentation of both absolute and relative effect sizes is recommended	NA
Ancillary analyses	18	Results of any other analyses performed, including subgroup analyses and adjusted analyses, distinguishing pre-specified from exploratory	NA
Harms	19	All important harms or unintended effects in each group (for specific guidance, see CONSORT for harms)	NA
Discussion			
Limitations	20	Trial limitations, addressing sources of potential bias, imprecision, and, if relevant, multiplicity of analyses	4
Generalisability	21	Generalisability (external validity, applicability) of the trial findings	4
Interpretation	22	Interpretation consistent with results, balancing benefits and harms, and considering other relevant evidence	3-4
Other information			
Registration	23	Registration number and name of trial registry	NA
Protocol	24	Where the full trial protocol can be accessed, if available	NA
Funding	25	Sources of funding and other support (such as the supply of drugs), the role of funders	NA

**Table 2:** Age, sex, BMI, duration of procedures, and temperatures LChol: laparoscopic cholecystectomy; LCol: laparoscopic colectomy; LCholw: LChol warmed; LCholrt: LChol room temperature; LColw: LCol warmed; LColrt: LCol room temperature; SD: standard deviation; BMI: body mass index

	LCholw n=20	LCholrt n=20	P-value
	Mean±SD	Mean±SD	
Age (years)	51.65±13.66	49.35±14.56	0.609
	49.30±12.38	52.35±14.43	0.478
BMI (kg/m^2^)	29.59±5.47	28.58±5.33	0.558
	28.36±5.39	27.07±4.83	0.43
Core temperature (^0^C)	36.47±0.74	35.34±0.76	0.001^*^
	36.32±0.63	35.16±0.99	0.001^*^
Axillary temperature (^0^C)	35.31±0.84	34.41±0.63	0.001^*^
	35.42±0.91	34.29±0.33	0.001^*^
Sex (Female)	15 (75.0%)	16 (80.0%)	1
	11 (55.0%)	10 (50.0%)	0.752

**Table 3:** VAS at follow-up times in laparoscopic colectomy patients VAS: visual analog scale; LCol: laparoscopic colectomy; LColw: LCol warmed; LColrt: LCol room temperature; SD: standard deviation

VAS value	LColw n=20	LColrt n=20	P-value
	Mean±SD (median)	Mean±SD (median)	
VAS at 30thminute	2.20±2.55 (0.00)	3.50±3.12 (5.00)	0.113
VAS at 1th hour	5.75±1.62 (6.00)	5.10±2.15 (5.00)	0.133
VAS at 6th hour	5.05±1.61 (5.00)	5.70±1.08 (6.00)	0.180
VAS at 12thhour	3.45±2.09 (4.00)	3.25±2.15 (4.00)	0.756
VAS at 24thhour	0.60±1.10 (0.00)	1.75±1.80 (2.00)	0.27

**Table 4:** VAS at follow-up times in LChol patients VAS: visual analog scale; LChol: laparoscopic cholecystectomy; LCholw: LChol warmed; LCholrt: LChol room temperature; SD: standard deviation

VAS value	LCholw n=20	LCholrt n=20	P-value
	Mean±SD (median)	Mean±SD (median)	
VAS at 30thminute	3.80±2.98 (4.00)	5.20±2.75 (6.00)	0.092
VAS at 1thhour	4.45±1.67 (5.00)	4.85±1.69 (4.50)	0.822
VAS at 6th hour	4.25±1.86 (4.50)	5.05±2.06 (5.00)	0.078
VAS at 12th hour	2.25±2.40 (1.50)	2.10±2.08 (2.00)	0.850
VAS at 24th hour	1.40±147 (2.00)	1.20±1.28 (1.00)	0.770

## Discussion

In the present study, we investigated the effect of an intraoperative warming heating blanket with forced air warming on postoperative pain and analgesic consumption in short and long laparoscopic surgical procedures. In the United Kingdom, the National Institute for Health and Care Excellence recommends the following: (i) monitoring patients' intraoperative temperature every 30 minutes; (ii) delaying anesthesia induction until the patient's body temperature rises above 36°C; (iii) warming intravenous fluids and blood products to 37°C; (iv) using a forced-air warming device placed over patients for heating purposes for procedures lasting longer than 30 minutes; and (v) if normothermia can be maintained through warmed irrigation or external warming devices, it can be concluded that there is no need for warmed insufflation systems and the additional costs they bring [3,4,7]. Herein, we planned this work with the hypothesis that hypothermia could be prevented through a patient warming procedure rather than warming and humidifying CO2 gas.

Sammour et al. [10] tested the hypothesis that warmed and humidified CO2 insufflation attenuates postoperative pain and improves healing by reducing peritoneal inflammation in laparoscopic colon surgery and found the surgery duration, gas volume used, and pneumoperitoneum duration were similar. There were no significant differences in intraoperative morphine consumption, postoperative recovery, or analgesic usage on the 1st, 2nd, and 3rd days. Therefore, they did not recommend utilizing warmed and humidified insufflation gas in laparoscopic colon surgery due to the lack of clinical benefit. In the present study, the insufflation durations were similar to those in this study, though the number of LCol was limited. In LColw and LCholw, the VAS score at the 24th hour was significantly lower. Of note is that the amount of analgesia required was also found to be lower. Mouton and colleagues [11] investigated the effectiveness of warmed gas, and postoperative VAS scores were revealed to be significantly lower without any difference in analgesic consumption. They also asserted that the intraoperative patient temperature was similar in both groups. However, we recognized a significantly higher core temperature in the group warmed with a device blowing warm air from the outside. In addition, the VAS scores in the early postoperative period in LColw and LCholw were insignificant.

Birch et al. [12] concluded in a systematic review conducted with randomized controlled trials that the use of warmed gas had no effect on intraoperative body temperature and did not impact total postoperative pain or analgesic requirements. Su and Nieh [13] expressed that a forced-air warming device increased the effectiveness of patient warming and reduced complications in cases undergoing laparoscopic surgery to hinder intraoperative hypothermia and its associated complications. We achieved normothermia in cases using an active forced-air warming device; thus, in the warmed study groups, patients had significantly lower pain scores at the 30th minute, 1st hour, and 6th hour. Schwenk et al. [14] emphasized that significant pain may arise in the early period after laparoscopic intervention. The severe pain experienced in the earlier period substantially improved pain and feeling well after 24 hours. In the present study, the VAS scores were significantly lower in the 24th hour, and there was a noticeable decrease in pain after 12 and 24 hours. Nevertheless, contrary to expectations, at 24 hours, 15% of the non-warmed colectomy cases had an analgesic requirement.

In a randomized controlled study conducted by Nguyen et al. [15], the effect of warming CO2 gas insufflated into the cases warmed with an external blanket on postoperative pain was investigated, and no significant difference was asserted. In a non-randomized clinical study investigating the effect of short-term warming of patients during the preoperative period in laparoscopic urological surgeries on postoperative complications, the warming patients attenuated emerging hypothermia, shivering, and pain. We did not record hypothermia in the warmed cases while they were using the identical forced-air heaters. As such, during the 1st and 6th hours, a significant reduction in pain in the warmed ones was observed without any difference in the subsequent hours. Despite some limitations, we can conclude that preoperative warming of patients prevents hypothermia and reduces postoperative complications [16].

Last but not least, a study conducted on pediatric patients who had undergone laparoscopic surgery demonstrated that heated and humidified CO2 insufflation better maintained the internal temperature than standard CO2 insufflation. They also propounded positive postoperative outcomes, such as reduced shivering and shorter hospital stays for patients [9,17]. Of note, pediatric cases face more significant challenges in maintaining their body temperature and are at a higher risk of hypothermia compared to adults. In the present study, normothermia was achieved using external heating devices. However, considering the difficulty of achieving this in the pediatric patient group, heating and humidifying CO2 gas might be beneficial.

Limitations

We encountered challenges in evaluating two different surgical patient groups in the same study, especially in terms of temperature and CO2 insufflation duration.

## Conclusions

In this study, we were unable to demonstrate a positive effect of patient warming on VAS. However, we found that external warmers can prevent hypothermia independently of surgical duration. Herewith, the association between patient warming, postoperative pain, and analgesic requirements still needs to be fully elucidated. Particularly in the aspects above, this issue merits further investigation.
